# Supplementary material for: L-tyrosine-bound ThiH structure reveals C–C bond break differences within radical SAM aromatic amino acid lyases
Source: Nat Commun. 2022 Apr 27;13:2284. doi: 10.1038/s41467-022-29980-4 (PMC9046217; doi:10.1038/s41467-022-29980-4)
Supplement: Supplementary file 1 — Supplementary information file [file 41467_2022_29980_MOESM1_ESM.pdf]

Supplementary Information for

L-tyrosine-bound ThiH structure reveals C-C bond break differences within radical SAM aromatic amino acid lyases

Patricia Amara,[1] Claire Saragaglia,[1] Jean-Marie Mouesca,[2] Lydie Martin[1] and Yvain Nicolet.[1,*]

[1]Univ. Grenoble Alpes, CEA, CNRS, IBS, Metalloproteins Unit, F-38000 Grenoble, France.

[2]Univ. Grenoble Alpes, CEA, CNRS, IRIG-DIESE-SyMMES-CAMPE, 38000 Grenoble, France

*corresponding author: yvain.nicolet@ibs.fr

Supplementary Methods

QM/MM calculations. Calculations were performed with programs from the Schrödinger suite[1] and in particular the QSite program for QM/MM potentials. Our starting models were the L-tyrosine bound *Tc*ThiH crystal structure (Supplementary Table 1) and the L-tryptophan bound *Sa*NosL crystal structure[2] (PDB ID 4R34). Hydrogen atoms were constructed and the protonation state of each residue was optimized using the Protein Preparation Protocol. The DFT method in the Jaguar program was used with the BH and HLYP functional[3] that partially takes non-dynamical electron correlation into account, with the 6-31G** basis set. The active sites' regions were treated quantum mechanically (see below) while the rest of the systems was treated with the OPLS2005 molecular mechanical (MM) force field[4]. Residue side-chains missing in the crystal structures and hydrogen atoms were built and the MM part as well as the residue protonation states were optimized, holding the QM parts. The systems were then geometry-optimized, constraining the atoms positions of residues lying over 12 Å away from the atoms belonging to the QM parts.

<u>L-tyrosine bound ThiH starting model (Supplementary Fig. 13a):</u>

Substrate modeling: In L-tyrosine, an amino group was modeled rather than an ammonium group due to its interaction with the R300 residue side-chain. Indeed, after geometry-optimization, an ammonium group facing R300 led to a large deviation from the crystal structure, and deprotonating the R300 side-chain led to proton transfer from the ammonium back to R300. In addition, the L-tyrosine carboxylate group was kept deprotonated as it interacts with the protonated E158 residue side-chain; both carboxylate groups deprotonated put them further apart and protonating the L-tyrosine carboxylate group led to a proton transfer to the E158 side-chain. L-tyrosine was initially modeled with a phenol group but it disrupted the four anion-dipole interactions observed in the crystal structure (Fig. 2c) while geometry-optimization of L-tyrosine, bound to ThiH, with a phenolate group (see main text) maintained the experimentally-observed anchoring in the protein. This unexpected depronotation of the phenol was confirmed with molecular dynamics (MD) simulations of L-tyrosine bound *Tc*ThiH (see below).

QM part: 5´-dA, L-tyrosine (substrate), 2 water molecules interacting with the substrate carboxylate group, residues Y80, E158, Y181, E183, R300 and Q336. The QM part consists of 137 atoms.

Radical 5´-dA•: The [5´-dA• + L-Tyr]-bound ThiH model that we call [L-Tyr-NH_2] throughout the manuscript was obtained by i) removing a hydrogen atom from the 5´-dA C5´H_3 (Fig. 1) in the L-tyrosine bound ThiH geometry-optimized structure and ii) geometry-optimizing the resulting system, called R (Supplementary Fig. 14c).

L-tryptophan bound NosL starting model (Supplementary Fig. 13b):

Substrate modeling: The L-tryptophan amino group was modeled rather than an ammonium group due to its interaction with R323 and the carboxylate group was kept deprotonated as previously determined[5].

QM part: 5´-dA, L-tryptophan (substrate), 3 water molecules interacting with the substrate carboxylate group (Fig. 2c), residues Y90, N175, E204, R323 and Q363. The QM part consists of 127 atoms.

Radical 5´-dA•: The model [5´-dA• + L-Trp]-bound NosL that we call [L-Trp-NH_2] throughout the manuscript was obtained by simply removing a hydrogen atom from the 5´-dA C5´H_3 (Supplementary Fig. 1) in the L-tryptophan bound NosL geometry-optimized structure and ii) geometry-optimizing the resulting system, called R (Supplementary Fig. 14c).

Reactant scan: The substrate amino group, N(H1)(H2), orientation was investigated starting from the models we obtained for ThiH and NosL (Supplementary Fig. 13) and by scanning the Cβ-Cα–N-H1 dihedral angle (Supplementary Fig. 14). The QM part was reduced to 5´-dA•, L-tyrosine (or L-tryptophan), E183 (or E204) and R300 (or R323). Single-energy points were obtained with the M06-2X density functional[6] and the cc-pVTZ(-f) basis set. We also used the latter functional and basis set for all single-energy point calculations below.

Hydrogen abstraction by 5´-dA•: To investigate the hydrogen atom transfer from the substrate (L-tyrosine or L-tryptophan) amino group to 5´-dA• (Fig. 1 and Supplementary Fig. 1), we i) scanned the hydrogen distance from the amino group nitrogen atom to the 5´-dA• C5´ atom and ii) searched for the transition state that was optimized. Vibrational frequency calculations were performed to check that all minima (R and P) had positive frequencies and that the transition states had only one imaginary

frequency. We further checked it was the relevant transition state for the reaction of interest by using the intrinsic reaction coordinate method (IRC). Starting points were the geometries of the 1st point on the scan for ThiH (R in red in Supplementary Fig. 14c) and of the 5th (productive) and 1st (non-productive) points for NosL (R and R´, respectively, in green in Supplementary Fig. 14c). Reactants (R_{IRC}) and products (P_{IRC}) geometries issued from the IRC calculations were further optimized leading to R_{opt} for ThiH and R_{opt} and $R´_{opt}$ for NosL. For ThiH, R_{IRC} and P_{IRC} (before optimization) were used for tunneling evaluation (see section below). As expected, R_{opt} (ThiH) ≈ R (within the calculation precision) and R_{opt} (NosL) ≈ R (NosL) and the corresponding products had the correct geometries. Single-energy points of the results are presented in Fig. 4. In $R´_{opt}$ (NosL) the L-tryptophan Cβ-Cα–N-H1 dihedral angle is at 23.8° on the flat potential region observed in the corresponding reactant scan (Supplementary Fig. 14b,c). The scan exhibits a small barrier going from the 1st to the 5th point. We searched for the transition state between the 1st and the 5th point and found a transition state at 4.6 kcal.mol^{-1} from the 1st point and at 0.6 kcal.mol^{-1} from the 5th point, explaining why going down from TS´ we did not reach the global minimum. We have already observed this haziness around the first steps of the NosL reaction[5]. Indeed, we previously investigated NosL Cα-C bond break starting from a model of [L-Trp-NH•] already assuming the hydrogen abstraction by 5´-dA•. We started from the same X-ray model as the present study. At the time we had suggested a thermally-activated rotation of the amino-bearing arm in order to weaken the Cα-C bond and then described the reaction of the Cα-C bond break as well as the recombination of the resulting •COO⁻ to the substrate ring. In the present work, we have looked in further details at the very first step of the reaction and we observe alternatively that the reactant global minimum is not the one allowing the enzymatic reaction to proceed. Thus, the previous amino-bearing arm rotation we had proposed now appears unnecessary, being here translated into the fact that the energy surfaces around the minima are flat in the case of NosL. This feature is actually most probably the one that differentiates NosL from ThiH.

Note: throughout the text, the dihedral called Φ refers to Cβ-Cα–N-H1 for the reactants [L-Tyr-NH_2] and [L-Trp-NH_2] models and Φ refers to Cβ-Cα–N-H for the transition states and the products (where H is the remaining hydrogen atom after hydrogen abstraction from -NH_2 by 5´-dA•). Also the H that is transferred will be called H_t.

MD simulations. Starting from the QM/MM geometry we obtained for L-tyrosine bound *Tc*ThiH (Supplementary Fig. 13a and Supplementary QM/MM calculations' section above), two models were constructed with the –OH group of L-tyrosine protonated or deprotonated, respectively. A third model was constructed from the [*p*-cresyl• + DHG]-ThiH model we obtained from QM/MM calculations of the reaction path, where the *p*-cresyl• was protonated to obtain *p*-cresol. In the Schrödinger suite, by default and for any force field, the charges of [Fe-S] clusters are formal, i.e., +2 or +3 for the Fe atoms and -2 for the inorganic bridging S atoms. From our experience, artifactual interactions can be encountered with this charge model[7]. In [Fe_4S_4] clusters, the iron ions exhibit local high spin configurations S = 5/2 (ferric ion) and S = 2 (ferrous ion). The cluster oxidized state consists of two mixed-valence iron pairs, (Fe^{II}-Fe^{III}) of spin S = 9/2 each, antiferromagnetically coupled, yielding a cluster with M_s=0. We used reduced charges of +0.4 and -0.4 on iron ions and inorganic sulfur ions, respectively[8]; the total charge of the resulting [Fe_4S_4] cluster is 0, thus the +2 charge of the oxidized state was spread over the originally negatively-charged ligands (Cβ et Sγ of cysteine ligands and the O and N atom of the methionine bound to the [Fe_4S_4] unique iron as in Fig. 2b). The OPLS3e force field[9] was employed. Na^+ or Cl^- ions were added to neutralize our models that were further solvated with a periodically replicated orthorhombic water box using the TIP4P water model. The resulting systems consisted of approximately 36,500 atoms. The Desmond program[10] included in the Schrödinger suite was used for molecular dynamics simulations. The default protocol was used to equilibrate the system and heat it from 0 to 333 K, the optimal growth temperature of *Thermosinus carboxydivorans*. The simulations of both L-tyrosine- and [*p*-cresol + DHG]- bound models were then run for 250 ns with a time step of 2 fs in the isobaric, isothermal (NPT) ensemble with a temperature of 333 K and a pressure of 1 atm.

QM calculations. To study the effect of dynamics at the ThiH active site, we extracted a sample of conformations from the deprotonated L-tyrosine bound ThiH MD trajectory at 32.5 ns, 105 ns, 149 ns, 150.75 ns, 160 ns, 180 ns, 194.5 ns and 245 ns (see *MD simulations*' section above). Since, in our QM/MM model of the reaction (1) the products, resulting from the Cα-Cβ bond break were not completely formed (see Fig. 5 and main text) and (2) dehydroglycine is tightly held by two residues (arginine 300 and glutamate 158), we tried to find conformations leaving more space for *p*-cresyl• to

separate from dehydroglycine. Thus, the frames we chose correspond to the largest distances between the substrate phenyl ring and tyrosine residues 76 and 181 (see Fig. 6b and main text). The minimal active site model consisted of L-Tyr, 5´-dA, Y76, P78, Y80, L126, L128, E158, Y181, E183, S298, T299, R300, S318, S321, T323, F337, I339 and the two water molecules interacting with the carboxylate group of L-Tyr (Fig. 2c). Cα and Cβ atoms of all residues except the substrate ones were held fixed during the L-tyrosine Cα-Cβ energy scans. For the 180-ns frame (solid black line in Fig. 6a) and the same QM model extracted for the QM/MM geometry of the reactant L-Tyr-NH• (Fig. 5 and red line with triangles in Fig. 6a), a QM geometry-optimization of the last point on the energy curve (at around 3.1 Å) was performed.

Tunnel effect evaluation. Given the fact that the calculated reaction barrier using QM/MM potential were relatively high (Fig. 4 and Supplementary Table 2) and that the associated transition states' frequencies were also high, we decided to quantify the impact of hydrogen tunneling on the energetic gain it provides for the hydrogen atom abstraction by 5´-dA• at the amino-nitrogen position of L-tyrosine or L-tryptophan, for ThiH and NosL, respectively.

There are quite a few different non-variational "rigid barrier" descriptions of the tunnel effect all based on the transition state[11–17]. Among them, Eckart[16] proposed an asymmetric potential barrier, later further explicated by others[17,18], which takes into account the energy difference between reactants and products. This model has been successfully used to estimate tunneling correction[19,20] even though it tends to overestimate the values of the tunneling factors below 300 K[21]. This class of models rely on the use of a well-defined - hence "rigid" or "non-variational" - transition state. Moreover, it is assumed that, in the neighborhood of this transition state, the motion along the direction of the one-dimensional reaction path can be separated from all other motions of the interacting species, thus neglecting contribution from the heavy-atom environment. As a consequence, a potential barrier can be defined along this path from reactants to products via a single hydrogen transfer reaction coordinate which is orthogonal to all other modes of motions of the interacting species. A more complete picture of hydrogen tunneling would therefore include contributions of motions from the heavy-atom environment and a number of quantum-mechanical theories have been proposed, calculating Arrhenius curves from first principles, including tunneling. These theories (*Small-Curvature Tunneling*, *Instanton*, etc…) start

with an *ab initio* calculation of the reaction surface (energies, gradients and hessians) before using either quantum or statistical rate theories in order to calculate appropriate rate constants[15,22–31].

Such involved calculations are however beyond the scope of our work. In effect, radical SAM enzyme structures all exhibit a tight proximity of 5´-dA (a good mimic of the highly reactive 5´-dA• radical) and the targeted substrate hydrogen to be abstracted[32]. This, along with the fact that even at high temperature MD simulations of L-tyrosine bound ThiH do not reveal drastic conformational changes around 5´-dA, justifies our choice of an analytic estimation of hydrogen tunneling effect using the Eckart approach (see below). Moreover, we will show that we already achieve, by following the current QM/MM minimum energy path approach, both a lowering of the effective barrier by a rough factor 2 and (first order) kinetic constant values (in s^{-1}) within the range observed in enzymatic systems (see Supplementary Table 4 and the reference cited there). All numerical applications will be based on the DFT models (BH and HLYP with 6-31G**) extracted from QM/MM results (see section above and Supplementary Table 2).

Along the minimal energy path (R → TS → P) defined by our QM/MM calculations, Eckart tunneling occurs when a hydrogen atom is transferred through the reaction barrier as a result of its wave-like property. Its description involves the estimation of a transmission coefficient (called *Q* below) which depends on i) the barrier height from reactants to transition states, ii) the barrier widths related to the imaginary frequency/curvature at the transition states; the higher the frequency, the narrower the barrier, the easier the tunneling, and iii) the mass of the transferred particle (1 a.u. for hydrogen).

Hydrogen tunneling is expected to lower the barrier from reactants R = amino group of substrate and 5´-dA• (NH_2 + •C5´H_2) to products P (•NH + C5´H_3) via the transition state TS (HN…H_t…C5´H, where H_t is the tunneling hydrogen).

We use the following potential barrier function[16,17]:

$$V(x) = \frac{AZ}{1+Z} + \frac{BZ}{(1+Z)^2} \quad \text{where } Z = \exp(2\pi x/\ell) \qquad \text{(eq. S1)}$$

The parameter *A* controls the asymptotic value for large positive *x* values (i.e. *A* = *P*-*R*; for *A* = 0, the curve becomes symmetric). The parameter *B* is related to the height of the *V*(*x*) potential curve (see below). Finally, ℓ (Å) corresponds to the range values of *x* (Å) for which *V*(*x*) varies significantly (roughly the base width of the potential).

The position of the maximum is given by:

$$x_m = \frac{1}{2\pi} \ln \frac{B+A}{B-A} \quad \text{(eq. S2)}$$

(x_m = 0 for A = 0) at which position

$$V(x_m) = E \equiv TS - R = \frac{(A+B)^2}{4B} \quad \text{(eq. S3)}$$

$V(x_m) = E$ is the barrier from R to TS. From Eq. S3:

$$B = 2E - A + 2\sqrt{E(E-A)} \quad \text{(eq. S4)}$$

For A = 0, B = 4E.

Before estimating tunnel corrections for NosL and ThiH, we need to calculate the second derivative of $V(x)$ at the maximum (TS) point (x_m, $V(x_m)$) to link it with the (imaginary) frequency value computed for TS. From eq. S1, at x_m:

$$|V''(x_m)| = (\pi/\ell)^2.(B/4).(1-y)^3.(1-y+2y^2) \quad \text{(eq. S5)}$$

where $y = A/B$ ($|y| < 1$). From eq. 2 in reference 12:

$$\nu = (1/2\pi).(V''(x_m)/m)^{1/2} \quad \text{(eq. S6)}$$

where m is the mass of the hydrogen atom. Numerically:

$$\nu\ (\text{cm}^{-1}) = 170.5.(B/m)^{1/2}.(1-y)^{3/2}.(1-y+2y^2)^{1/2}/\ell \quad \text{(eq. S7)}$$

with B in kcal/mol, m in a.u. (for hydrogen : m = 1) and ℓ in Å.

From DFT (QM/MM) values in Supplementary Table 2, one derives the parameter values in Supplementary Table 3 reported in the corresponding Supplementary Fig. 18.

We can now compute tunnel corrections for NosL and ThiH. From reference 17, the discriminating parameter for the Eckart model is:

$$C = h^2/(8m\ell^2) \equiv 0.476/m\ell^2 \text{ (in kcal.mol}^{-1}\text{; } \ell \text{ in Å)} \quad \text{(eq. S8)}$$

from which the following quantities are derived:

$$\alpha = \frac{1}{2}\sqrt{W/C},\ \beta = \frac{1}{2}\sqrt{(W-A)/C},\ \delta = \frac{1}{2}\sqrt{(B-C)/C} \quad \text{(eq. S9)}$$

(W is a generic energy integration variable used below). With ℓ values around 0.66 Å, C values are around 1.07 kcal.mol^{-1}. The criteria $B > C$ (i.e. δ real; cf. Eq. S9) is satisfied[17]. As a consequence, the permeability is given by (eq. 6 in reference 17):

$$G = \frac{\cosh\{2\pi(\alpha+\beta)\} - \cosh\{2\pi(\alpha-\beta)\}}{\cosh\{2\pi(\alpha+\beta)\} + \cosh\{2\pi\delta\}} \quad \text{(eq. S10)}$$

To obtain the transmission coefficient Q (quantum tunneling along the reaction coordinate : eq. 4 in reference 12), we integrated the following equation:

$Q = \exp(E/kT).\int_0^{\infty}(1/kT).\exp(-W/kT).G(W).\mathrm{d}W$ (eq. S11)

Finally, the reaction rate coefficient $k(T)$ is given by:

$k(T) = Q.(kT/h).\exp(-E/RT)$ (eq. S12)

In turn, the correcting factor Q can be translated into an equivalent lowering of the barrier (from eq. S12):

$k(T) = (kT/h).\exp(-E^{eff}/RT) \rightarrow E^{\mathrm{eff}} = E - RT.\ln(Q)$ (eq. S13)

where R is the molar gas constant (1.9872 10^{-3} kcal.K^{-1}.mol^{-1}) and k is in s^{-1}. Numerically:

$E^{eff} = E - 0.593\ (T/298).\ \ln(Q)$ (kcal.mol^{-1} ; T in Kelvin) (eq. S14)

The calculated tunneling corrections and reaction rate constants $k(T)$ are reported in Supplementary Table 4 (60°C and 25°C for *Thermosinus carboxydivorans* and *Streptomyces actuosus*, respectively) but also represented as a function of temperature in Supplementary Fig. 19 to take into account the optimal bacterial growth. The tunnel corrections are quantitatively significant: E_{eff}/E ~50 % for ThiH and ~60 % for both NosL cases.

To check the above procedure, more specifically the internal consistency between the two energetic Eckart parameters A, B on the one hand and the internal length scale ℓ (computed from the frequency v: Supplementary Table 2,3) on the other hand, we turn briefly to structural data pertaining to IRC states. We thus computed IRC points for ThiH (as an example) starting from TS and sliding both toward R (two points: R_{IRC} and intR = intermediate between TS and R) and toward P (two points: P_{IRC} and intP = intermediate between TS and P). This procedure results into five points along the IRC path (R_{IRC}, intR, TS, intP and P_{IRC}). Then, and for the sake of consistency, we measured N-H_t (called p), C5´-H_t (called q) and N-C5´ (called r) distances for these five points along the IRC path (cf. values reported in Supplementary Table 5 and accompanying scheme). IRC distances (p, q, r) from Supplementary Table 5 allow us to compute IRC x coordinates (cf. Eq. S1) centered on TS (set at x(IRC) = 0.0 Å). From R to TS, we selected p(rel.) distances, and from TS to P, we selected q(rel.) distances ('rel.' refers to a value calculated relative to that of TS).

Data from Supplementary Table 5 allow us to report the IRC points on the ThiH Eckart potential curve (cf. Supplementary Fig. 18-20). It can be seen from Supplementary Fig. 20 that IRC points are close enough to the Eckart potential curve derived from QM/MM data and that IRC internal coordinates x(IRC) as defined in Supplementary Table 5 are therefore compatible with the ℓ value (0.654 Å) derived from the QM/MM frequency at TS. Thus, combining energetic parameters extracted from the catalytic reaction (Supplementary Tables 2,3) with structural data collected along the IRC path (Supplementary Table 5) shows the consistency of the Eckart model as applied to our ThiH and NosL data. In particular, it allows us to validate *a posteriori* the fact that, in both cases, hydrogen transfer occurs along very short distances as a consequence of the local tight control exerted on both 5´-dA• radical and substrates by the local respective protein matrices.

Supplementary Figures

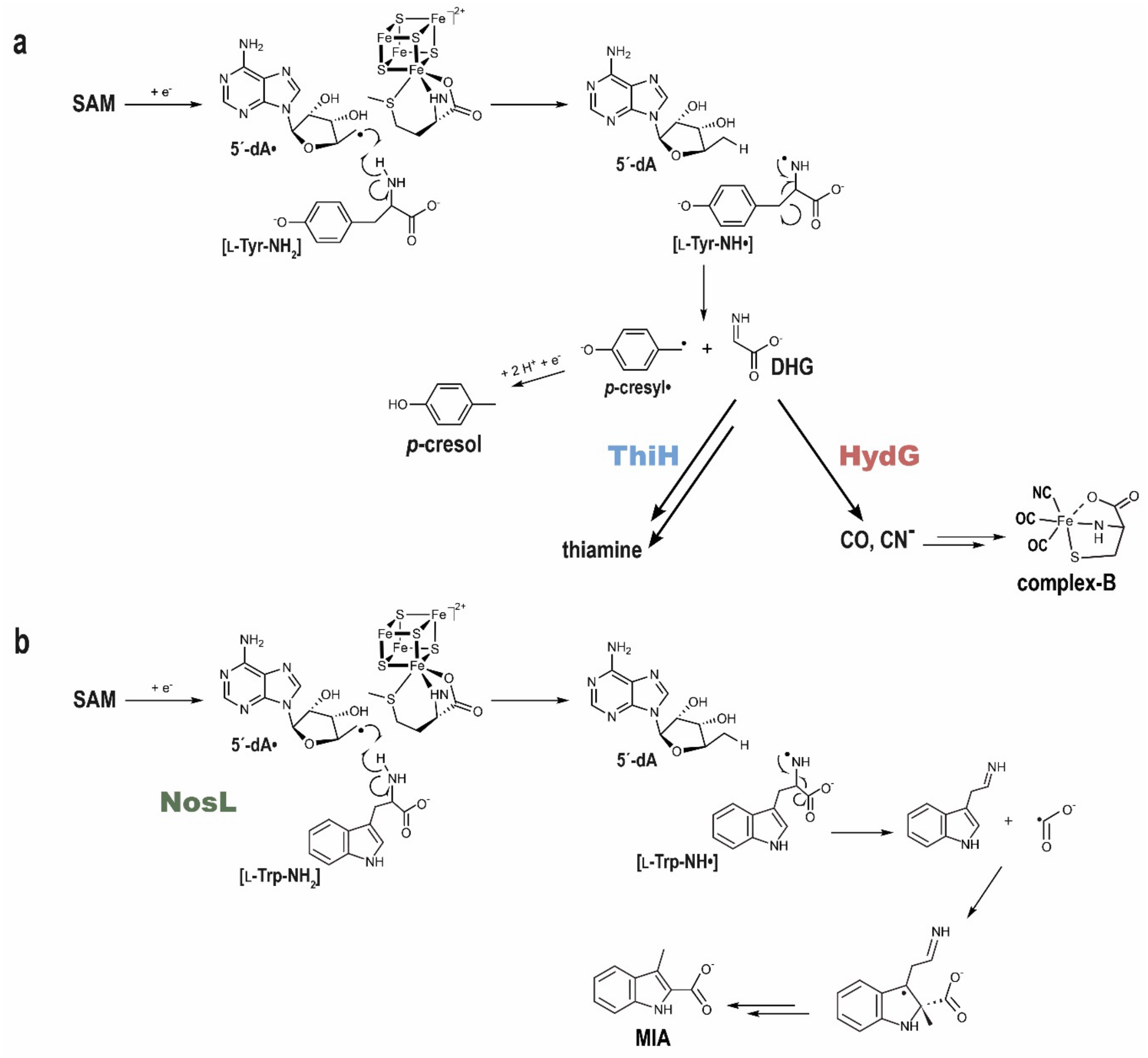

Supplementary Fig. 1 | Reactions catalyzed by **a**, ThiH and HydG and **b**, NosL.

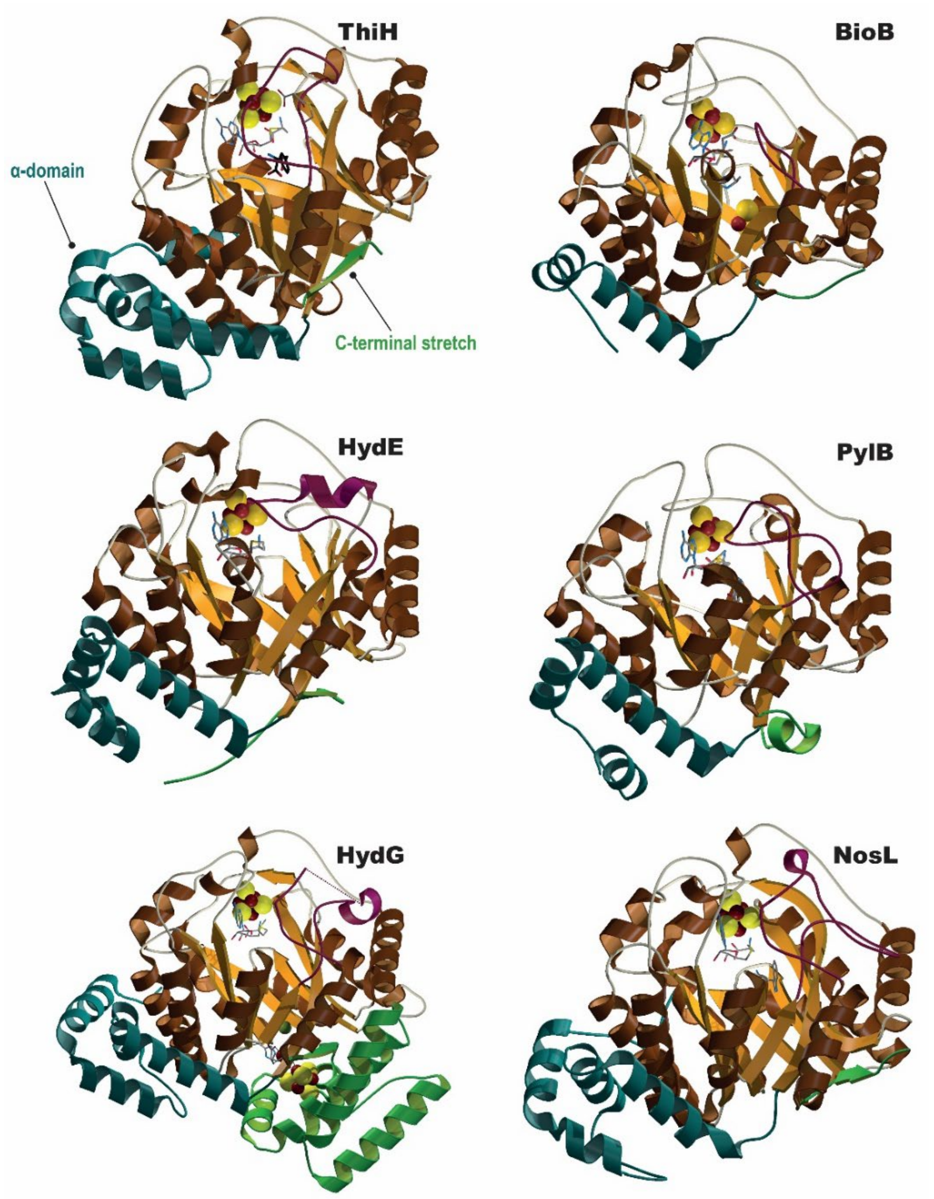

Supplementary Fig. 2 | Structural comparison between *Tc*ThiH (this work), BioB from *Escherichia coli* (PDB ID 1R30[33]), *Tm*HydE (PDB code 3CIW[34]), PylB from *Methanosarcina barkeri* (PDB ID 3T7V[35]), *Ti*HydG (PDB ID 4WCX[36]) and *Sa*NosL (PDB ID 4R34[2]). The N-terminal domain is in blue, helices and strands for the TIM barrel are depicted in brown and goldenrod, respectively; the C-terminal stretch is in green. The variable loop between strand S8 and helix H8 is depicted in burgundy. For all protein structures, the [Fe_4S_4] cluster and the SAM (or SAH or 5´-dA) are represented as van der Waals spheres and sticks, respectively with standard atom colors. The substrate of ThiH (L-tyrosine) and NosL (L-tryptophan) and the product of PylB are also represented as sticks. For HydG, the auxiliary cluster of HydG and its ligands are represented as van der Waals sphere and sticks respectively and a chloride ion is represented as a green sphere.

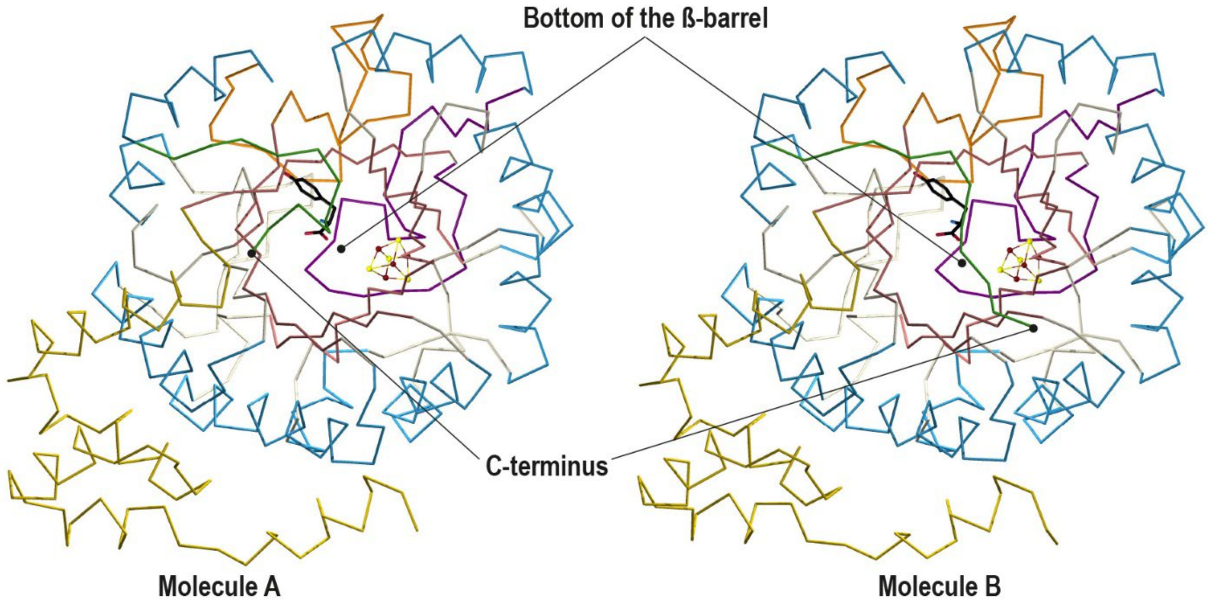

Supplementary Fig. 3 | Cα-trace representation of molecules A (left) and B (right) of the *Tc*ThiH refined model where the radical SAM core is depicted in indian red (β-strands) and blue (α-helices). The N- and C-terminal stretches are depicted in gold and green, respectively. The flexibility of the C-terminal stretch is shown by the very different conformations it adopts in molecules A and B (see main text). This is reminiscent of what was recently discussed for the radical SAM enzyme HydE[37].

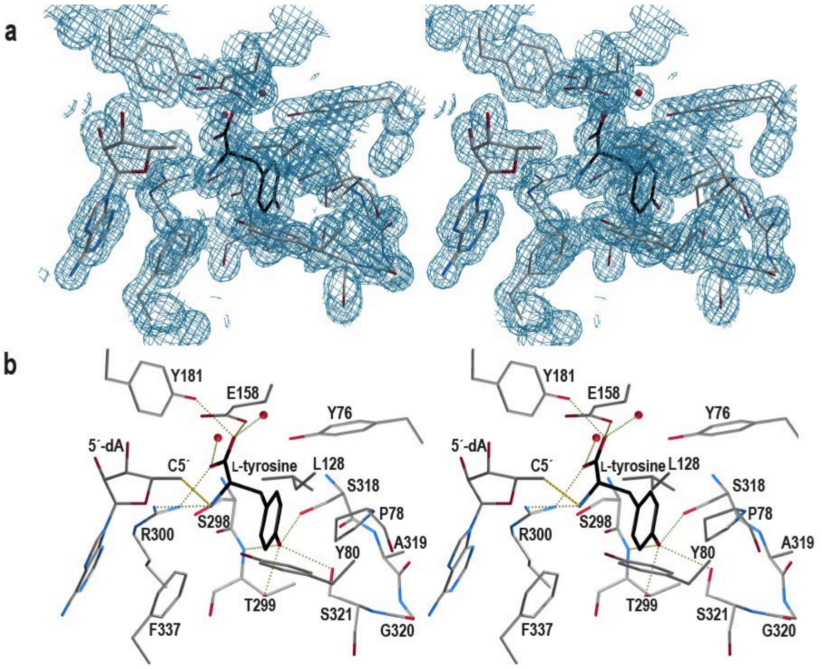

Supplementary Fig. 4 | a, Substrate (L-tyrosine) binding mode at the *Tc*ThiH active site with the *2Fo−Fc* electron density map for L-tyrosine and it environment contoured at 1 σ and depicted as steel blue mesh (cover radius 2.0 Å); see also Fig. 2c. **b**, Stereoview of Fig. 2c. Substrate (L-tyrosine) binding mode at the *Tc*ThiH active site.

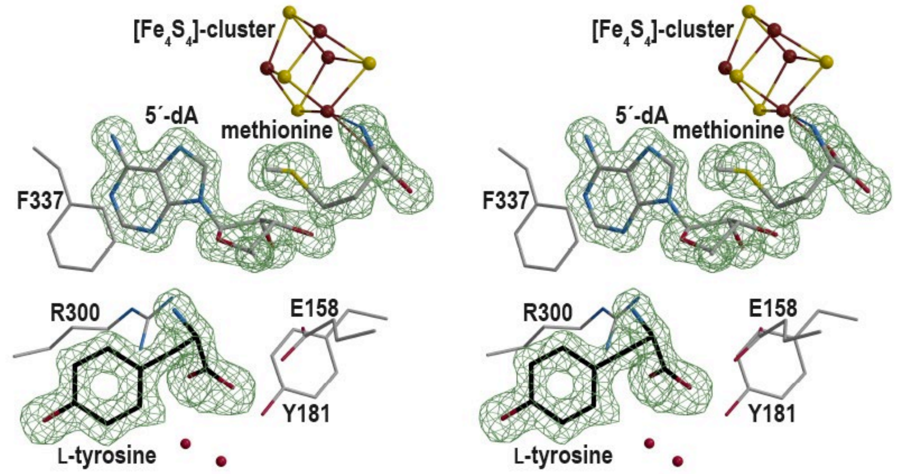

Supplementary Fig. 5 | Stereoview of Fig. 2b. Zoom of the L-tyrosine *Tc*ThiH binding site. The *Fo−Fc* difference Fourier (omit) electron density map for 5´-dA, L-methionine and L-tyrosine is contoured at 4.5 σ and depicted as green mesh (cover radius 2.0 Å).

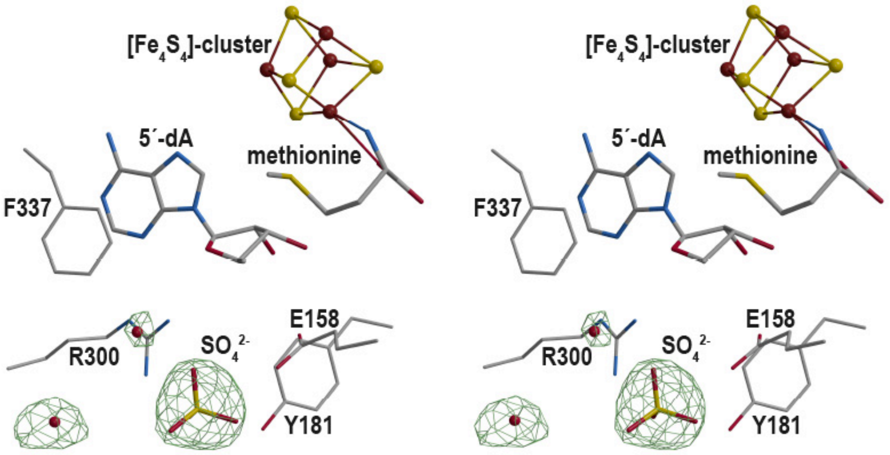

Supplementary Fig. 6 | Stereoview of Fig. 2e. Zoom of the substrate-free *Tc*ThiH active site in the same orientation as in Supplementary Fig. 5. The *Fo−Fc* difference Fourier (omit) electron density map surrounding the sulfate ion and the water molecules is contoured at 3 σ and depicted as green mesh (cover radius 2.0 Å).

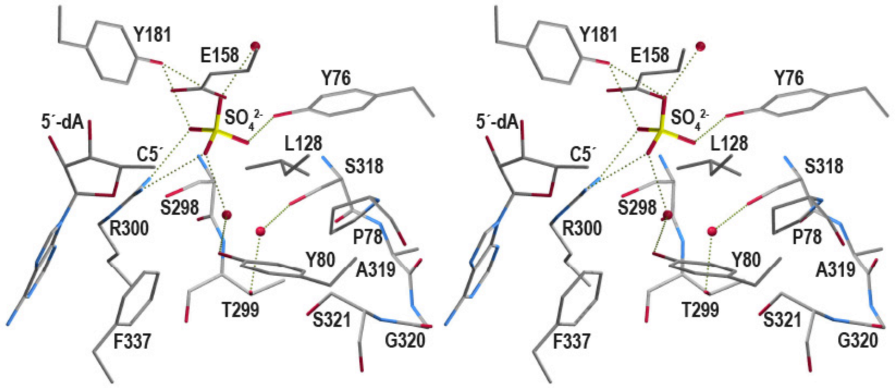

Supplementary Fig. 7 | Stereoview of Fig. 2f. Substrate-free *Tc*ThiH active site in the same orientation as in Supplementary Fig 4b.

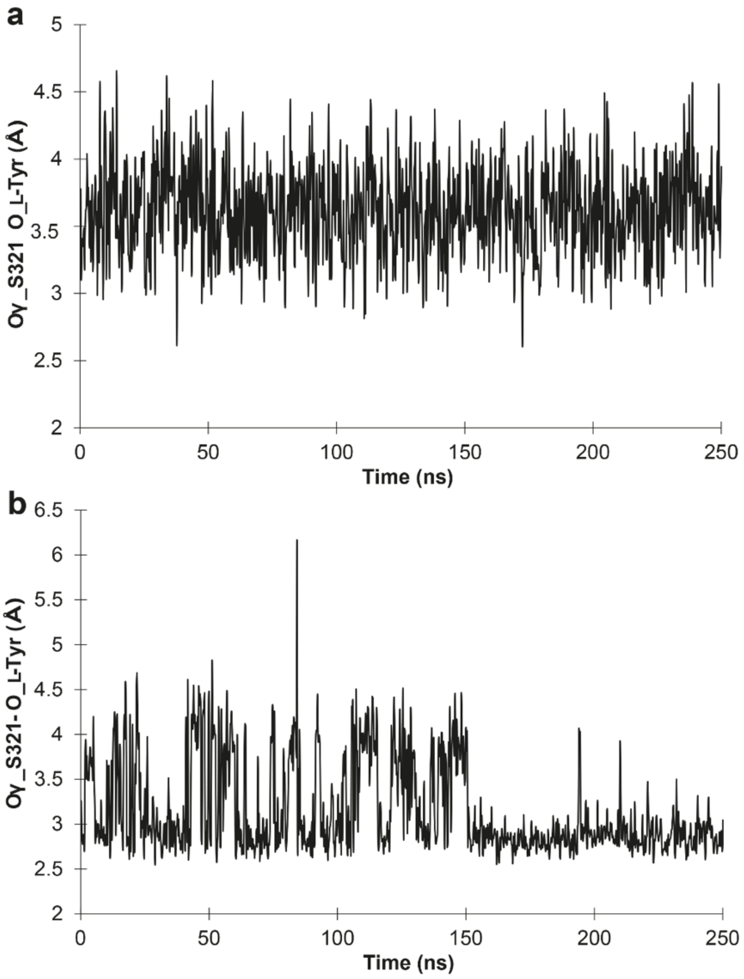

Supplementary Fig. 8 | Distance in Å between S321 Oγ and **a**, the L-tyrosine phenol oxygen atom and **b**, the L-tyrosine phenolate oxygen atom along a 250-ns MD simulation of L-tyrosine bound *Tc*ThiH at 333 K with substrate protonated or deprotonated, respectively (see Supplementary Methods and main text). Only the interactions with S321 and L-tyrosine are shown since the other three with T299 and S318 (Fig. 2d) are maintained throughout both MD simulations.

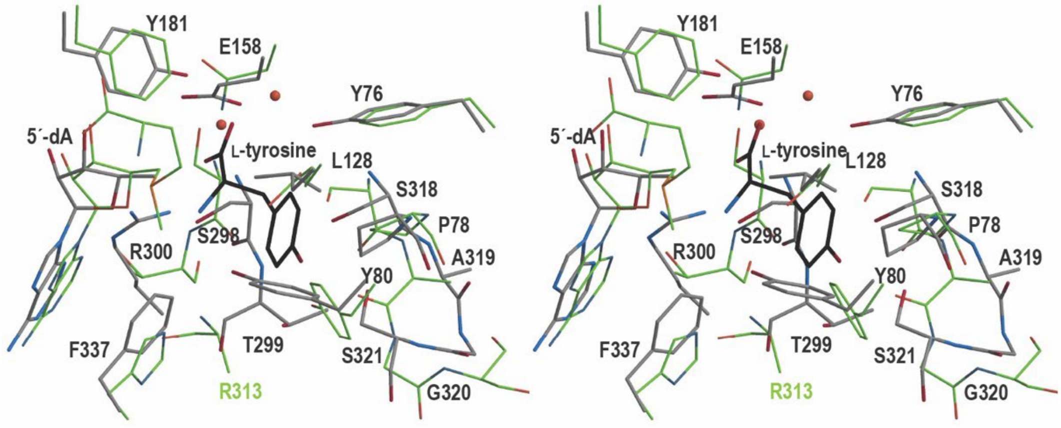

Supplementary Fig. 9 | Stereoview of the superposition of the L-tyrosine-bound *Tc*ThiH structure (zoom a the substrate binding site, carbon atoms in grey, this work) with the corresponding region in *Ti*HydG[36] (carbon atoms in green). The residues at the active site are represented as sticks with atoms other than carbon atoms represented with standard colors. Structural water molecules are represented as red spheres. Because in *Ti*HydG, the arginine residue R313 equivalent to R300 in *Tc*ThiH is at a very different position, it is indicated in green. All the other labels correspond to the residues in *Tc*ThiH.

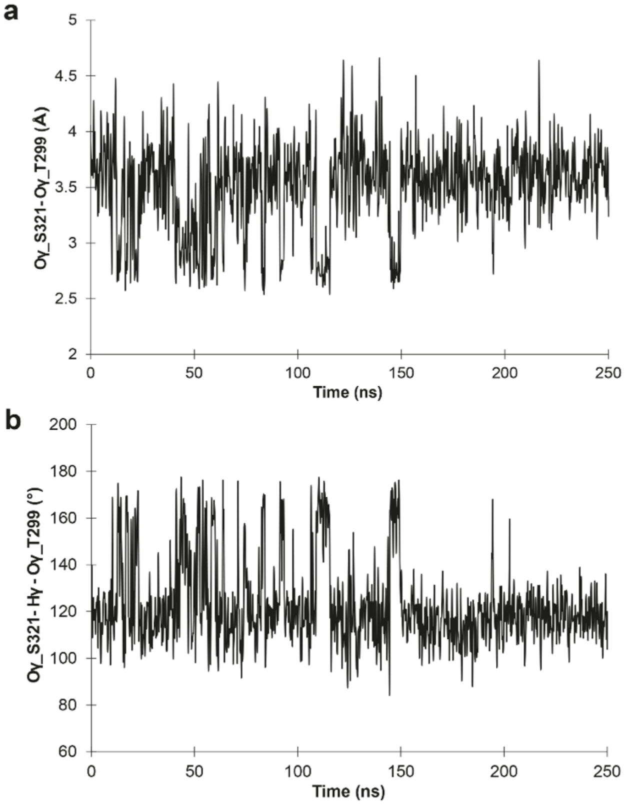

Supplementary Fig. 10 | a, Distance in Å between the S321- and T299- Oγ atoms and **b**, corresponding $O\gamma_{321}$-$H\gamma_{321}$-$O\gamma_{299}$ angle along the 250-ns MD simulation of L-tyrosine bound *Tc*ThiH at 333 K with a deprotonated substrate phenol group.

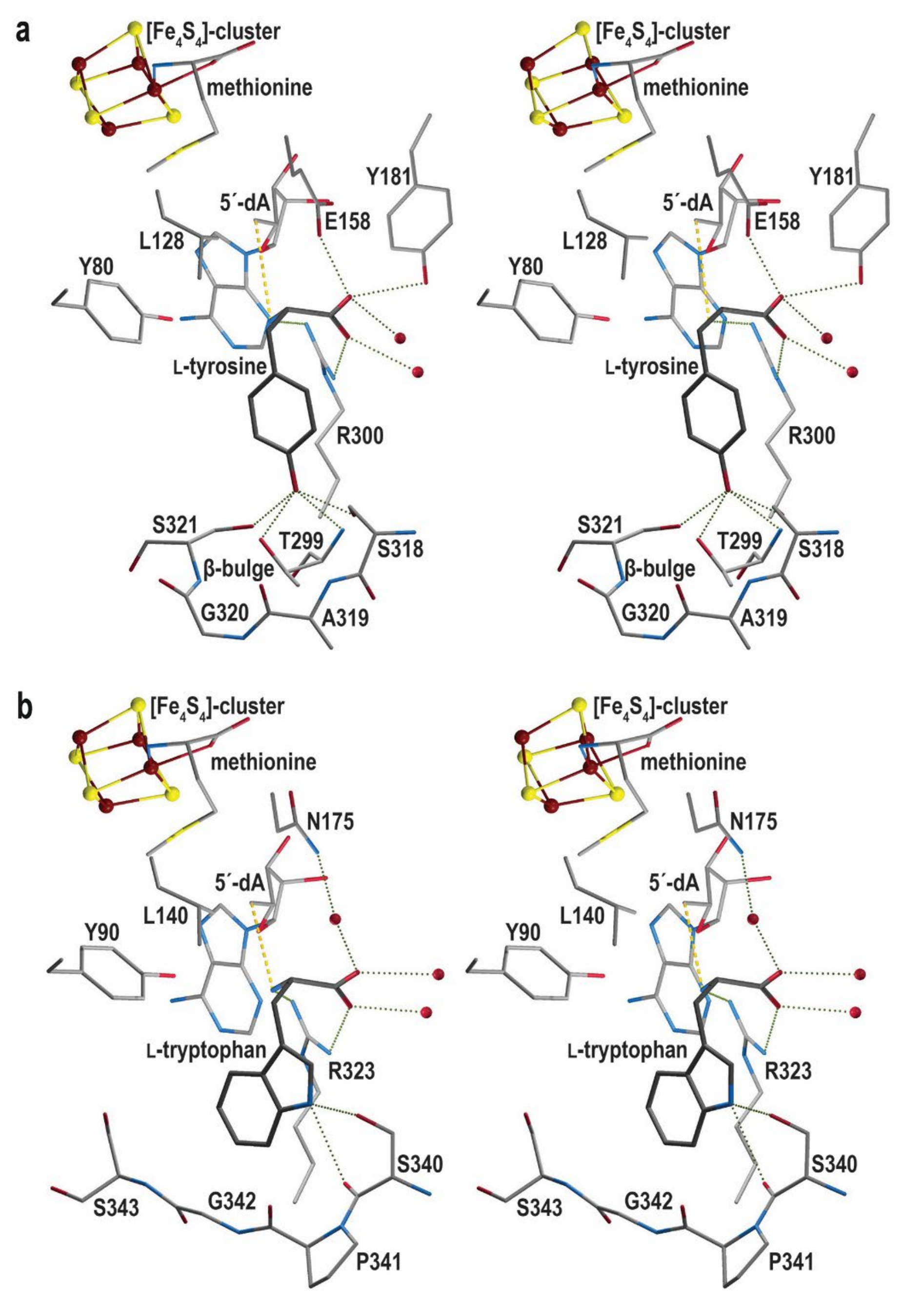

Supplementary Fig. 11 | Stereoview of Fig. 3b. Binding mode of **a**, L-tyrosine and **b**, L-tryptophan (right) in *Tc*ThiH (this work) and *Sa*NosL, respectively.

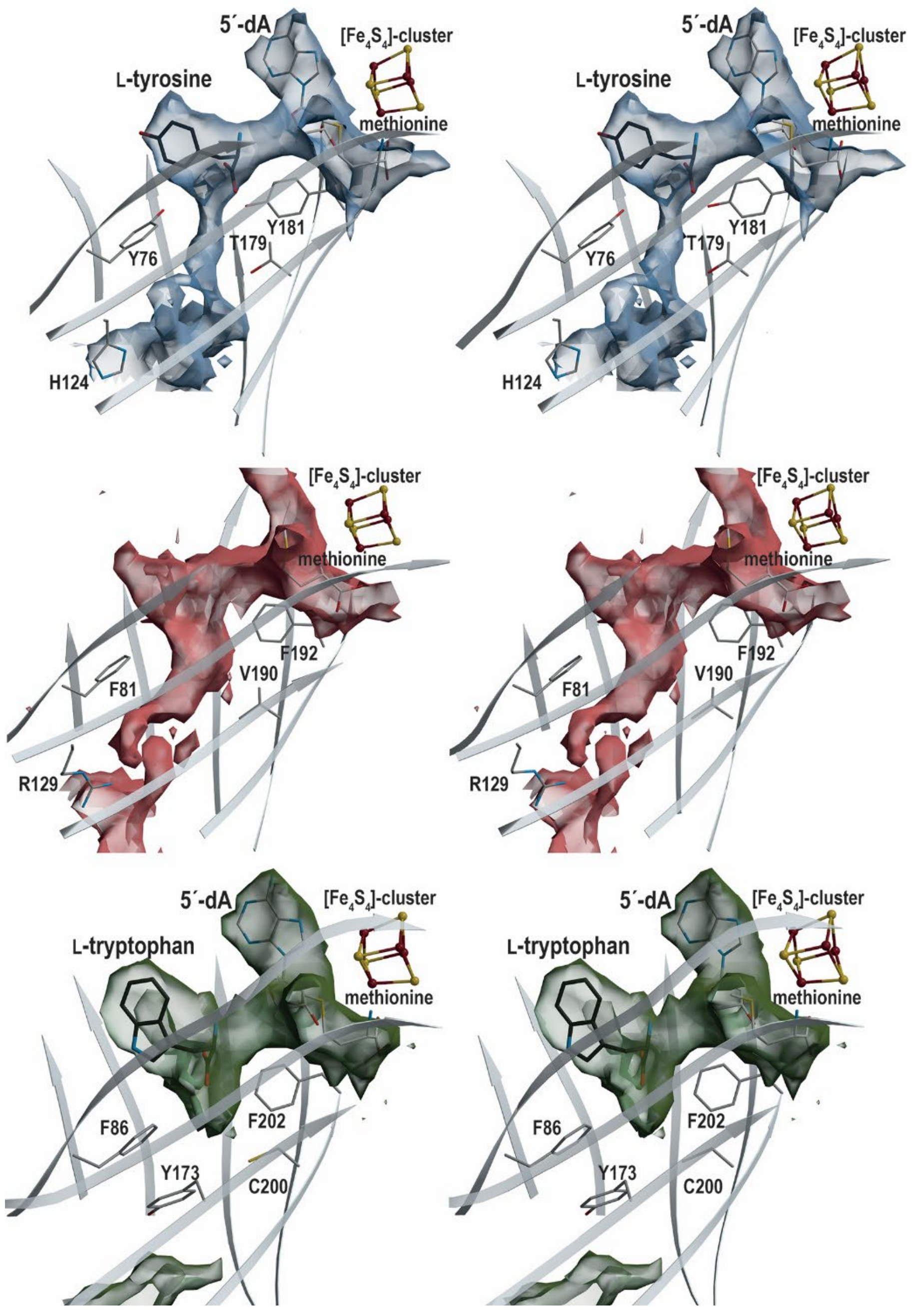

Supplementary Fig. 12 | Stereoview of Fig. 3c. The cavity maps, contoured with an accessible probe radius of 1 Å, were calculated using the X-ray models of *Tc*ThiH (top, steel blue), *Ti*HydG (middle, ruby-red) and *Sa*NosL (bottom, olive color). When present in the structural model, 5´-dA, the L-methionine bound to the [Fe_4S_4] cluster and the substrate were removed for the cavity calculation.

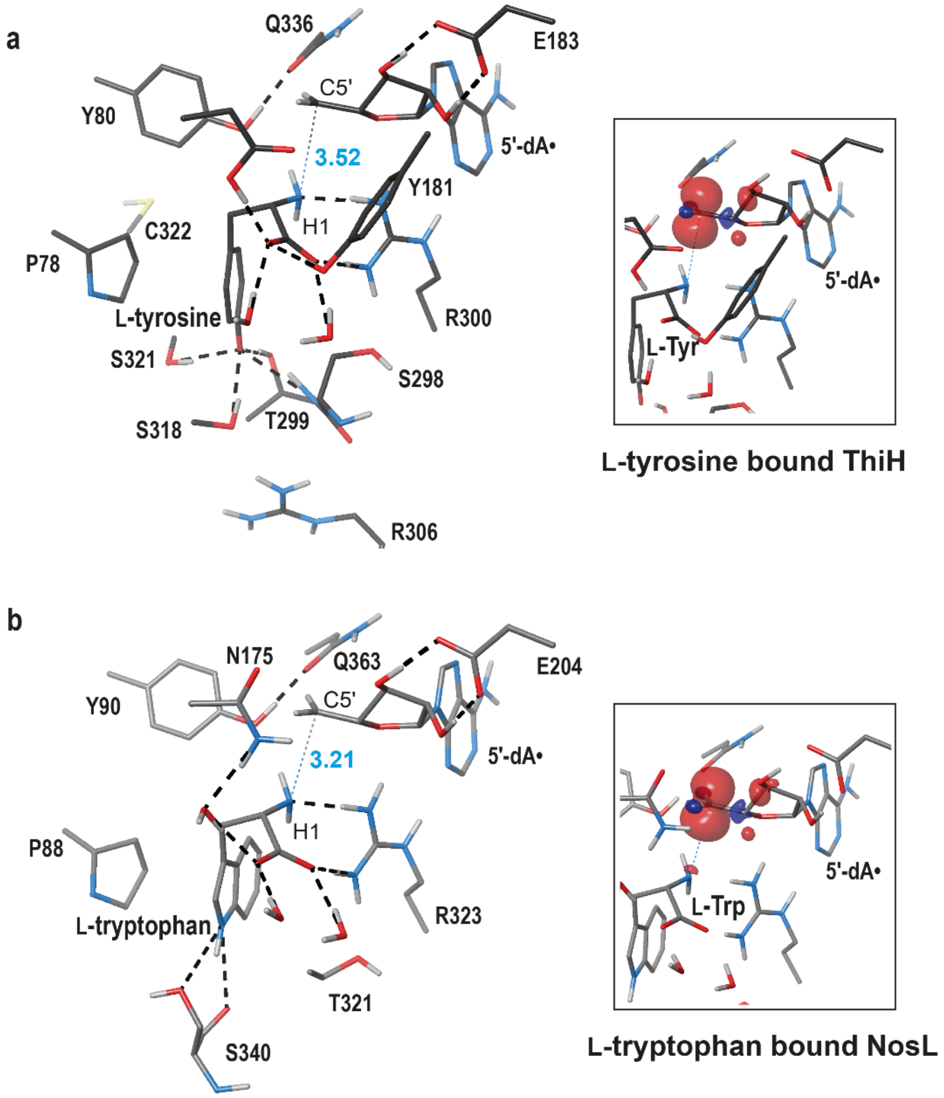

Supplementary Fig. 13 | Starting models of substrate-bound **a**, ThiH and **b**, NosL for QM/MM reaction path calculations. The model of L-tyrosine bound ThiH was also used for MD simulations. In the inserts, close-up views of the [L-Tyr-NH_2] and [L-Trp-NH_2] models are shown with the spin densities mainly located at the 5´-dA• C5´ atom. Only the substrate-binding site is represented (sticks) with C, N, O, S and H atoms represented in gray, blue, red, yellow and light gray, respectively; except for the 5´-dA C5´ atom, only polar hydrogens are shown for clarity. The H1 atom of the substrates' amino group is indicated as it is used in the dihedral scans of Supplementary Fig. 14.

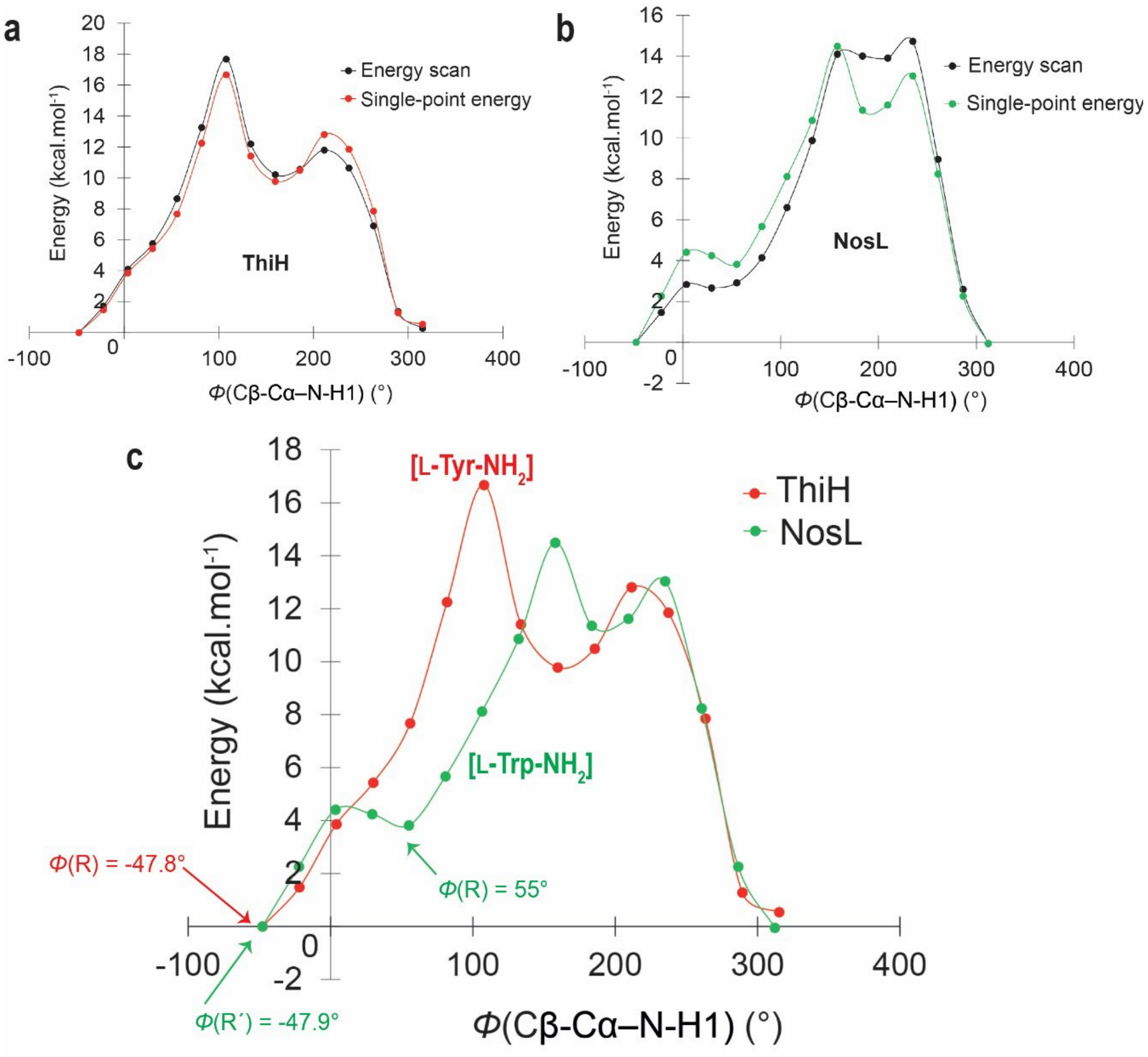

Supplementary Fig. 14 | Cβ-Cα–N-H1 dihedral scan **a**, in [L-Tyr-NH_2] (ThiH starting model in Supplementary Fig. 13a) and **b**, in [L-Trp-NH_2] (NosL starting model in Supplementary Fig. 13b). In **a** and **b**, the energy scans are represented as black lines with black circles. The corresponding single-energy points are represented as a red line with red circles and a green line with green circles for ThiH and NosL, respectively. Both curves are compared in **c**. In both cases, we are interested in energetically-reachable minima to study the hydrogen abstraction steps leading to [L-Tyr-NH•] and [L-Trp-NH•] (see Supplementary Fig. 1); these minima are indicated by their Φ angles in **c** (in red and green, for ThiH and NosL, respectively).

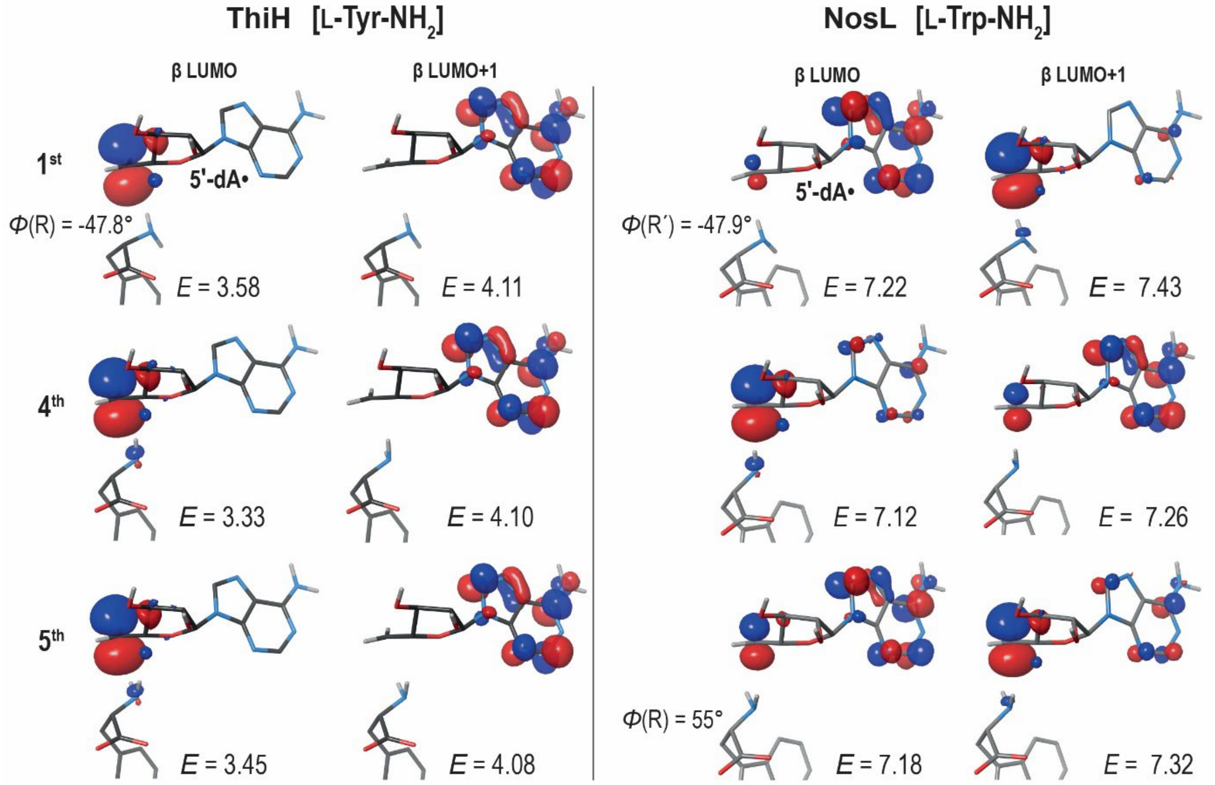

Supplementary Fig. 15 | Orbital analysis of the 1st, 4th and 5th single-energy points of the reactant scans ([L-Tyr-NH_2] and [L-Trp-NH_2] in Supplementary Fig. 14c). Lowest unoccupied molecular orbital (LUMO) and LUMO+1 representations for ThiH (left) and NosL (right) show an orbital crossing in the case of NosL while the corresponding orbitals are clearly separated in ThiH (see main text). For ThiH and NosL, orbital energies are reported in eV and the minima we chose to study the hydrogen abstraction (one for ThiH and two for NosL) are indicated by the corresponding value of their Cβ-Cα–N-H1 dihedral angles (Supplementary Fig. 14c). Note that the choice of the LUMO and LUMO+1 orbitals for the analysis has previously been justified[29].

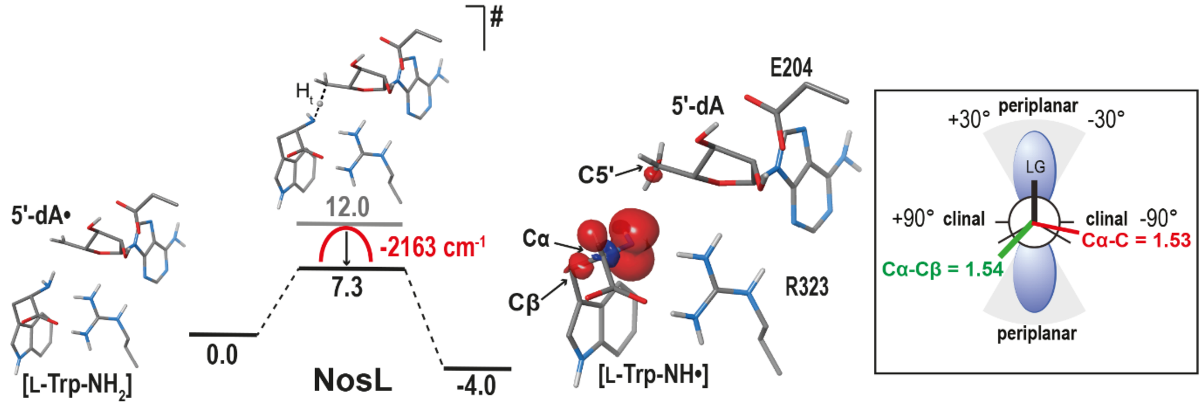

Supplementary Fig. 16 | Non-productive substrate hydrogen abstraction by 5´-dA• in *Sa*NosL. Reaction energy profile for the attack by 5´-dA•, leading to the formation of [L-Trp-NH•] from the non-productive reactant R´ (Φ = -47.9°, see main text and Supplementary Fig. 14) within the *Sa*NosL active site. Energies for the reactant and product are given in kcal.mol^{-1}; the TS energy in gray corresponds to the calculated value from the IRC calculations while the one in black is the barrier taking the tunneling effect into account (see main text and Supplementary Methods for details); the value of the TS single imaginary frequency is indicated in red; for the reactant R'_{opt}, transition state and the product P'_{opt} (Supplementary Table 2), a minimal model is included, represented as sticks with C, N, O and H atoms in gray, blue, red and light gray, respectively; the hydrogen atom that is transferred is represented as a light-gray ball in the transition state; the spin density is shown for the product. In the insert on the right: Newman representation of the dihedral angle (adapted from reference 39) between the partially occupied p orbital (blue) at the nitrogen radical center and the Cα-Cβ bond (in green) or the Cα-C bond (in red), where *p*-cresyl• (or •COO$^-$) is the leaving group (LG). The periplanar region is indicated in gray, the rest being clinal. Although not optimal, the orbital overlap allows the Cα-Cβ bond break while the Cα-C bond break is not possible with the dihedral angle clearly lying in the clinal region.

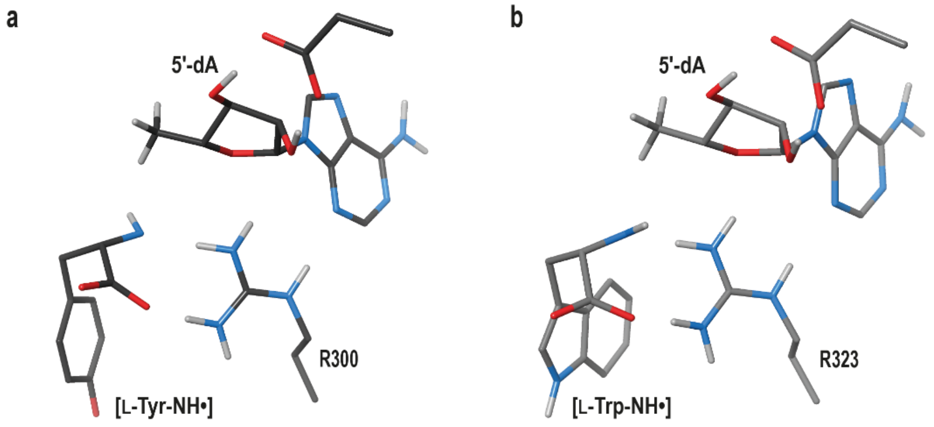

Supplementary Fig. 17 | Structures of **a**, [L-Tyr-NH•] and **b**, [L-Trp-NH•] using QM/MM potentials. Only a minimal model of the active site extracted from the corresponding QM/MM geometries is represented as sticks with C, N, O and H atoms in gray, blue, red and light gray, respectively; only polar hydrogens are shown for clarity.

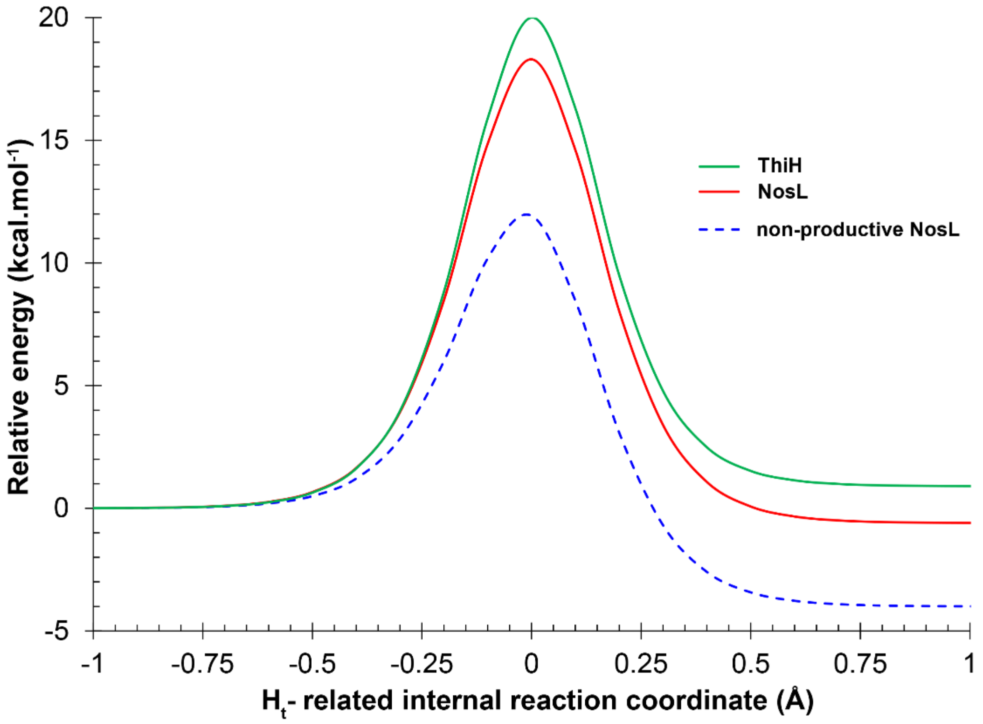

Supplementary Fig. 18 | Asymmetric Eckart barrier potentials for *Tc*ThiH R (green), *Sa*NosL productive R (red) and *Sa*NosL non-productive R´ (blue). All necessary *A* and *B* parameters' values are taken from Supplementary Tables 2,3.

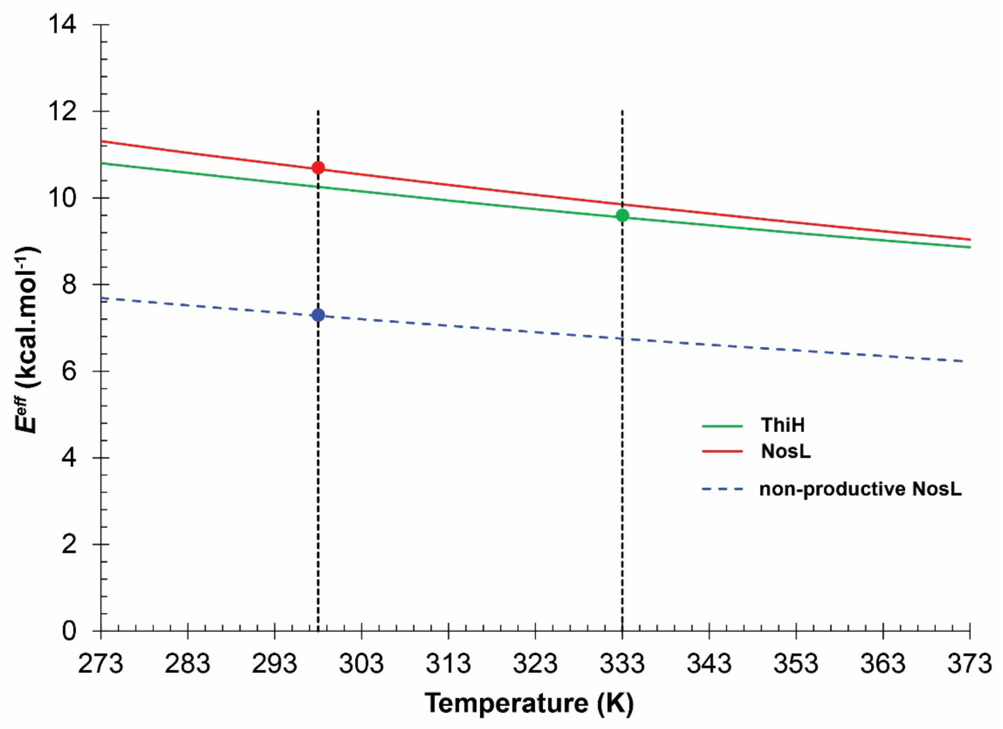

Supplementary Fig. 19 | Effective barriers derived from the Eckart model as a function of temperature for *Tc*ThiH R (green) and for both productive R (red) and non-productive R´ (blue) *Sa*NosL. Optimal bacterial growth temperatures at 333 K and 298 K for *Thermosinus carboxydivorans* and *Streptomyces actuosus*, respectively have also been reported (dashed vertical lines).

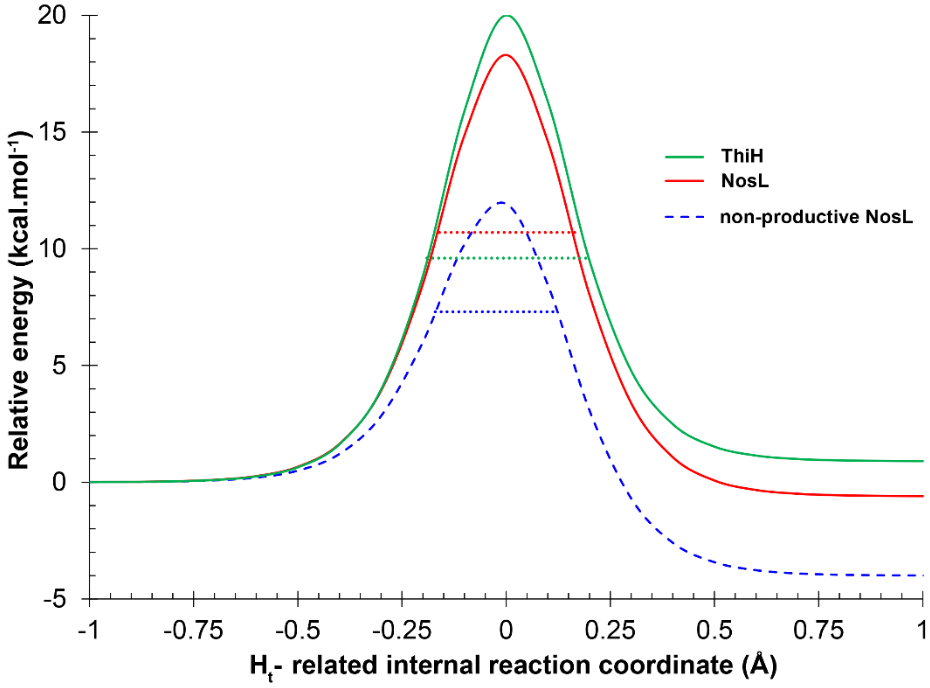

Supplementary Fig. 20 | Eckart barrier potentials for *Tc*ThiH R (green), *Sa*NosL productive R (red) and *Sa*NosL non-productive R´ (blue): same as in Supplementary Fig. 18. All necessary *A* and *B* parameters' values are taken from Supplementary Tables 2,3. Horizontal dotted lines now indicate E_{eff} effective barrier values (last column of Supplementary Table 4). Notice that the three effective barriers are close to one another (within 4 kcal.mol^{-1}) whereas the transition states (maxima of the profiles) lie within 8 kcal.mol^{-1}.

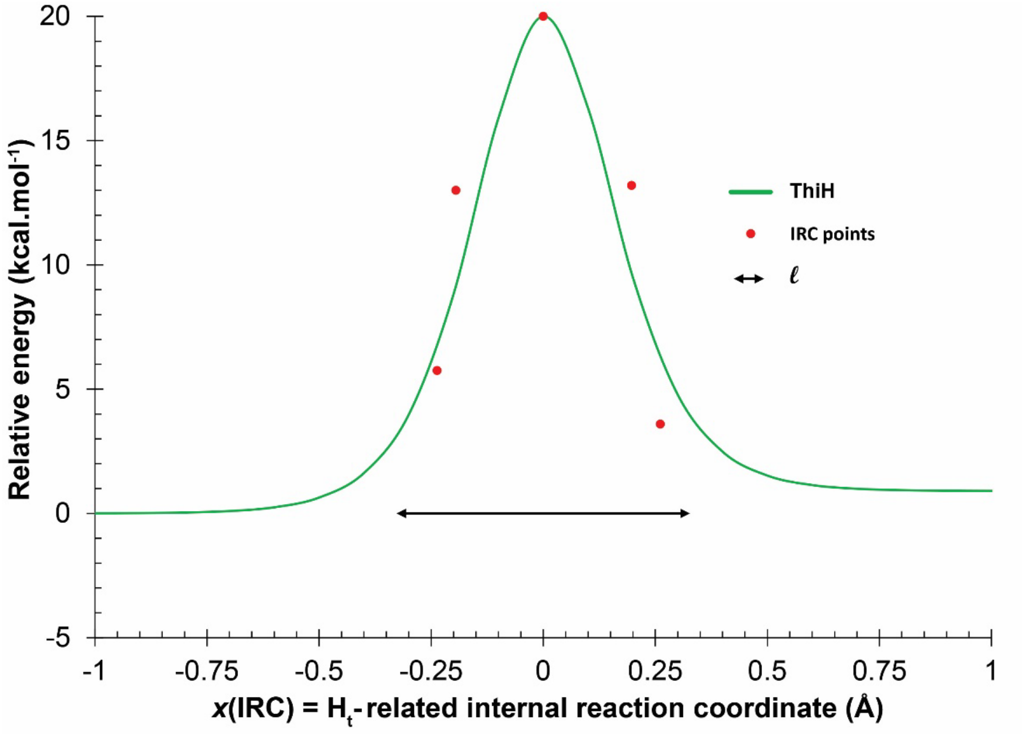

Supplementary Fig. 21 | Eckart potential profile for *Tc*ThiH (in green, same as in Supplementary Fig. 18, 20). IRC red points computed from the TS state are extracted from Supplementary Table 5. The extent of ***ℓ***, one of the three parameters of the Eckart model, for *Tc*ThiH corresponding to a value of 0.654 Å, is shown as a black double arrow.

Supplementary Tables

Supplementary Table 1 | Data processing and refinement statistics. Values in parentheses correspond to the highest resolution shell.

Dataset	L-tyrosine–bound *Tc*ThiH	Free–*Tc*ThiH
PDB code	**7PD1**	**7PD2**
Data collection		
Beamline	PROXIMA-1	PROXIMA-2A
Cell parameters (Å & °)	175.09 48.85 85.41 90 97.02 90	175.09 49.33 85.21 90 96.91 90
Space group	*C*2	*C*2
Wavelength (Å)	0.9785	1.000
Resolution range (Å)	47.03 – 1.27 (1.35 – 1.27)	47.46 – 1.99 (2.04 – 1.98)
R_{sym}	0.081 (0.947)	0.21 (2.051)
I/σ	11.56 (1.68)	6.11 (0.73)
CC(1/2) (%)	99.8 (78.4)	99.3 (23.0)
Completeness (%)	99.9 (97.9)	97.0 (89.0)
Multiplicity	6.8 (6.8)	6.1 (5.6)
Refinement statistics		
R_{work}	0.153	0.231
R_{free}	0.174	0.27
Nb of Reflections	188808	48713
Rmsd Bonds (Å)	0.012	0.004
Rmsd Angles (°)	1.29	0.56
Number of non-hydrogen atoms		
Protein	6043	5833
Ligands	101	100
Solvent	967	247
Average B-factor (Å^2)		
Protein	19.87	43.25
Ligand	25.34	52.63
Solvent	34.21	44.6
Ramachandran plot (%)		
Favored	98.22	97.94
Allowed	1.23	1.65
Outlier	0.62	0.41

Supplementary Table 2 | Single-energy points of the geometry-optimized IRC reactants, transition states and products for ThiH and NosL hydrogen abstraction reaction by 5´-dA• (Supplementary Methods and Fig. 4 and Supplementary Fig. 16). These values are those injected in the Eckart model and used in Supplementary Fig. 18, 20-21 to model the barrier potentials of *Tc*ThiH, *Sa*NosL productive and non-productive reactants (R), transition states (TS) and products (P). Energy values in black and parentheses are the values relative to the corresponding reactant global minima of ThiH and NosL (Supplementary Fig. 14c). We report (in red) values of the frequency *v* (cm^{-1}) computed for each TS. We also report structural parameters involved in hydrogen tunneling evaluation, namely N-C5´, NH_t ('t' stands for transferred) and $H_tC5´$ distances, as well as *Θ* (N-H_t-C5´) and *Φ* (Cβ-Cα–N-H (H1 for Rs')). Angles and dihedral angles are given in degrees.

	states *v* (cm^{-1})	*E* ($kcal.mol^{-1}$)	N-C5'	NH_t	H_tC5'	*Θ*	*Φ*
ThiH	$R_{opt} \approx R$<br>TS 2239<br>$P_{opt} \approx P$	0.0 (1.0)<br>20.0 (21.0)<br>0.9 (1.9)	3.35<br>2.59<br>3.29	1.01<br>1.25<br>2.29	2.91<br>1.34<br>1.08	107.0<br>179.6<br>153.7	-46.6<br>20.9<br>39.6
NosL*	$R_{opt} \approx R$<br>TS 2234<br>P	0.0 (4.4)<br>18.3 (22.7)<br>-0.6 (3.8)	3.03<br>2.55<br>3.09	1.01<br>1.26<br>2.32	2.52<br>1.34<br>1.08	111.1<br>158.4<br>127.1	58.1<br>-162.0<br>174.5
NosL	$R´_{opt} \neq R´$<br>TS´ 2163<br>P´	0.0 (5.1)<br>12.0 (17.1)<br>-4.0 (1.1)	3.01<br>2.57<br>3.17	1.01<br>1.24<br>2.17	2.02<br>1.34<br>1.08	164.3<br>174.7<br>152.8	23.8<br>30.4<br>46.0

* Values corresponding to the productive reactant (see main text and Fig. 4b).

Supplementary Table 3 | Frequency values *v* (cm^{-1}), *B* values derived from Eq. S4 (Tunneling section in Supplementary Methods), *A* values defined as *P-R* (cf. Supplementary Table 2), *y=A/B* ratios used in Eq. S5 and S7, x_m (*x* coordinate for the maximum of Eckart profile: cf. Eq. S2) and *ℓ* values derived from Eq. S7, all computed for the *Tc*ThiH and productive and non-productive *Sa*NosL.

	v (cm^{-1})	*B* ($kcal.mol^{-1}$)	*A=P-R* ($kcal.mol^{-1}$)	*y=A/B* (ratio)	x_m (Å)	*ℓ* (Å)
ThiH	2239	78.2	+0.9	+0.0115	+0.0024	0.654
NosL*	2234	74.4	-0.6	-0.0081	-0.0017	0.669
NosL	2163	55.7	-4.0	-0.0718	-0.0155	0.679

* Values corresponding to the productive reactant (see main text and Fig. 4b).

Supplementary Table 4. Barrier heights *E=TS-R* (kcal.mol^{-1}), estimations for tunnel corrections (column *–RT*.ln(*Q*) in kcal.mol^{-1}; cf. Eqs. S13-14) and effective barriers E^{eff} (kcal.mol^{-1}) computed for ThiH and NosL (productive R & non-productive R´). For non-productive NosL R´, an additional lowering by 0.7 kcal.mol^{-1} would be required (cf. Supplementary Table 2: difference between reactant values 5.1 and 4.4 kcal.mol^{-1} for NosL R´ and R, respectively). Tunnel length corresponds to the lengths of the horizontal dotted lines drawn in Supplementary Fig. 20. The average tunnel length value (0.34 Å) is about half the average ℓ value (0.67 Å = ~ width at the base of the Eckart profiles; see Supplementary Table 2).

	E=TS-R (kcal.mol^{-1})	*–RT*.ln(*Q*) (kcal.mol^{-1})	Tunnel length (Å)	E^{eff} (kcal.mol^{-1})	*k*(*T*)** (s^{-1})
ThiH (60°C)	20.0	-10.4	~0.39	9.6	3.5 10^6
NosL* (25°C)	18.3	-7.6	~0.33	10.7	9.0 10^4
NosL (25°C)	12.0	-4.7	~0.29	7.3	2.8 10^7

* Values corresponding to the productive reactant (see main text and Fig. 4b). **k(T) values, calculated at the optimal bacterial growth temperature, can be compared to those typically observed for first order enzymatic systems[40].

Supplementary Table 5 | N-H_t, H_t-C5´ and N-C5´ distances as well as N-H_t-C5´ angles (cf. *Θ* Scheme below) measured for ThiH for (QM/MM) IRC states: R_{IRC}, TS and P_{IRC} states as well as for two intermediate IRC states intR and intP. These five points follow the IRC path from TS toward both R on the one side and P on the other side. Are also computed *p* values relative to that of TS (*p*(rel.)), *q* values relative to that of TS (*q*(rel.)) and derived *x*(IRC) values selected from *p*(rel.) and *q*(rel.). *x*(IRC) values, spanning a distance of 0.5 Å, and corresponding energies (kcal.mol^{-1}) are used to plot IRC points (red) in Supplementary Fig. 21.

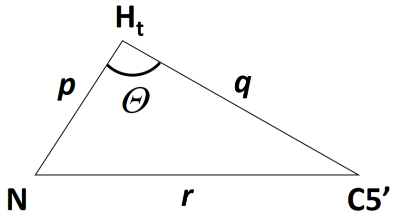

states	energies	*p* (N-H_t)	*q* (H_t-C5´)	*r* (N-C5´)	*Θ*	*p*(rel.)	*q*(rel.)	*x*(IRC) (Å)
R_{IRC}	5.8	1.013	1.963	2.974	175.2	-0.237	-0.623	-0.237
intR	13.0	1.055	1.595	2.650	179.4	-0.195	-0.255	-0.195
TS	20.0	1.250	1.340	2.590	179.6	0.000	0.000	0.000
intP	13.2	1.525	1.143	2.668	178.4	0.275	0.197	0.197
P_{IRC}	3.6	1.967	1.079	3.039	171.8	0.717	0.261	0.261

Supplementary References

1. Schrödinger Release 2020-4: Schrödinger, LLC, New York, NY, 2020.
2. Nicolet, Y., Zeppieri, L., Amara, P. & Fontecilla-Camps, J. C. Crystal structure of tryptophan lyase (NosL): evidence for radical formation at the amino group of tryptophan. *Angew. Chem. Int. Ed. Engl.* **53**, 11840–11844 (2014).
3. Aitken, H. M., Hancock, A. N. & Schiesser, C. H. Understanding (the lack of) homolytic substitution chemistry of sulfones. *Chem. Commun. (Camb)* **48**, 8326–8328 (2012).
4. Jorgensen, W. L., Maxwell, D. S. & Tirado-Rives, J. Development and testing of the OPLS all-atom force field on conformational energetics and properties of organic liquids. *J. Am. Chem. Soc.* **118**, 11225–11236 (1996).
5. Amara, P. *et al.* Radical *S*-Adenosyl-L-methionine tryptophan lyase (NosL): How the Protein Controls the Carboxyl Radical $\bullet CO_2^-$ Migration. *J. Am. Chem. Soc.* **140**, 16661–16668 (2018).
6. Zhao, Y. & Truhlar, D. G. Exploring the limit of accuracy of the global hybrid meta density functional for main-group thermochemistry, kinetics, and noncovalent interactions. *J. Chem. Theory Comput.* **4**, 1849–1868 (2008).
7. Crack, J. C. *et al.* Electron and proton transfers modulate DNA binding by the transcription regulator RsrR. *J. Am. Chem. Soc.* **142**, 5104–5116 (2020).
8. Mouesca, J.-M., Chen, J. L., Noodleman, L., Bashford, D. & Case, D. A. Density functional/Poisson-Boltzmann calculations of redox potentials for iron-sulfur clusters. *J. Am. Chem. Soc.* **116**, 11898–11914 (1994).

9. Roos, K. *et al.* OPLS3e: Extending force field coverage for drug-like small Molecules. *J. Chem. Theory Comput.* **15**, 1863–1874 (2019).
10. Desmond Molecular Dynamics System,. D. E. Shaw Research, New York, NY, 2020. Maestro-Desmond Interoperability Tools, Schrödinger, New York, NY, 2020.
11. Wigner, E. Über das Überschreiten von Potentialschwellen bei chemischen Reaktionen. *Zeitschrift für Physikalische Chemie* **19B**, 203–216 (1932).
12. Bell, R. P. The tunnel effect correction for parabolic potential barriers. *Trans. Faraday Soc.* **55**, 1–4 (1959).
13. Braun, J. *et al.* NMR Study of the tautomerism of porphyrin including the kinetic HH/HD/DD isotope effects in the liquid and the solid state. *J. Am. Chem. Soc.* **116**, 6593–6604 (1994).
14. Langer, U. *et al.* 15N NMR study of proton localization and proton transfer thermodynamics and kinetics in polycrystalline porphycene. *J. Phys. Org. Chem.* **13**, 23–34 (2000).
15. Limbach, H.-H., Miguel Lopez, J. & Kohen, A. Arrhenius curves of hydrogen transfers: tunnel effects, isotope effects and effects of pre-equilibria. *Philos. Trans. R. Soc. B: Biol. Sci.* **361**, 1399–1415 (2006).
16. Eckart, C. The penetration of a potential barrier by electrons. *Phys. Rev.* **35**, 1303–1309 (1930).
17. Bell, R. P. & Hinshelwood, C. N. The application of quantum mechanics to chemical kinetics. *Proc. R. Soc. Lond. Series A, Containing Papers of a Mathematical and Physical Character* **139**, 466–474 (1933).
18. Christov, S. G. Verallgemeinerte Näherungsausdrücke für die Durchlässigkeit von Potentialschwellen. *Annalen der Physik* **467**, 20–31 (1963).

19. Espinosa-García, J., Olivares del Valle, F. J. & Corchado, J. C. Transition state theory and Eckart's tunnelling factor: a good approximation for the calculation of bimolecular rate constants? *Chem. Phys.* **183**, 95–100 (1994).

20. Sandala, G. M., Smith, D. M., Coote, M. L., Golding, B. T. & Radom, L. Insights into the hydrogen-abstraction reactions of Diol Dehydratase: relevance to the catalytic mechanism and suicide inactivation. *J. Am. Chem. Soc.* **128**, 3433–3444 (2006).

21. Kuppermann, A. & Truhlar, D. G. Exact tunneling calculations. *J. Am. Chem. Soc.* **93**, 1840–1851 (1971).

22. Miller, W. H. Semiclassical limit of quantum mechanical transition state theory for nonseparable systems. *J. Chem. Phys.* **62**, 1899–1906 (1975).

23. Callan, C. G. & Coleman, S. Fate of the false vacuum. II. First quantum corrections. *Phys. Rev. D* **16**, 1762–1768 (1977).

24. Skodje, R. T., Truhlar, D. G. & Garrett, B. C. A general small-curvature approximation for transition-state-theory transmission coefficients. *J. Phys. Chem.* **85**, 3019–3023 (1981).

25. Kuznetsov, A. M. & Ulstrup, J. Proton and hydrogen atom tunneling in hydrolytic and redox enzyme catalysis. *Canadian J. Chem.* **77**, 1085–1086 (2011).

26. Fernández-Ramos, A., Miller, J. A., Klippenstein, S. J. & Truhlar, D. G. Modeling the kinetics of bimolecular reactions. *Chem. Rev.* **106**, 4518–4584 (2006).

27. Klinman, J. P. The widespread occurrence of enzymatic hydrogen tunneling, andits unique properties, lead to a new physical model for the origins of enzyme catalysis. *Procedia Chem.* **3**, 291–305 (2011).

28. Klinman, J. P. & Kohen, A. Hydrogen tunneling links protein dynamics to enzyme catalysis. *Annu. Rev. Biochem.* **82**, 471–496 (2013).

29. Layfield, J. P. & Hammes-Schiffer, S. Hydrogen tunneling in enzymes and biomimetic models. *Chem. Rev.* **114**, 3466–3494 (2014).

30. Bao, J. L. & Truhlar, D. G. Variational transition state theory: theoretical framework and recent developments. *Chem. Soc. Rev.* **46**, 7548–7596 (2017).

31. Meisner, J. & Kästner, J. Dual-level approach to Instanton theory. *J. Chem. Theory Comput.* **14**, 1865–1872 (2018).

32. Nicolet, Y. Structure–function relationships of radical SAM enzymes. *Nat. Catal.* **3**, 337–350 (2020).

33. Berkovitch, F., Nicolet, Y., Wan, J. T., Jarrett, J. T. & Drennan, C. L. Crystal structure of Biotin Synthase, an *S*-adenosylmethionine-dependent radical enzyme. *Science* **303**, 76–79 (2004).

34. Nicolet, Y. *et al.* X-ray structure of the [FeFe]-hydrogenase maturase HydE from *Thermotoga maritima*. *J. Biol. Chem.* **283**, 18861–18872 (2008).

35. Quitterer, F., List, A., Eisenreich, W., Bacher, A. & Groll, M. Crystal structure of methylornithine synthase (PylB): insights into the pyrrolysine biosynthesis. *Angew. Chem. Int. Ed. Engl.* **51**, 1339–1342 (2012).

36. Dinis, P. *et al.* X-ray crystallographic and EPR spectroscopic analysis of HydG, a maturase in [FeFe]-hydrogenase H-cluster assembly. *Proc. Natl. Acad. Sci. U.S. A.* **112**, 1362–1367 (2015).

37. Rohac, R. *et al.* Crystal structure of the [FeFe]-hydrogenase maturase HydE bound to complex-B. *J. Am. Chem. Soc.* **143**, 8499–8508 (2021).

38. Rohac, R. *et al.* Carbon-sulfur bond-forming reaction catalysed by the radical SAM enzyme HydE. *Nat. Chem.* **8**, 491–500 (2016).

39. Ruszczycky, M. W. & Liu, H.-W. Mechanistic enzymology of the radical SAM enzyme DesII. *Isr. J. Chem.* **55**, 315–324 (2015).

40. Radzicka, A. & Wolfenden, R. A proficient enzyme. *Science* **267**, 90–93 (1995).
