# Supplementary material for: L-tyrosine-bound ThiH structure reveals C–C bond break differences within radical SAM aromatic amino acid lyases
Source: Nat Commun. 2022 Apr 27;13:2284. doi: 10.1038/s41467-022-29980-4 (PMC9046217; doi:10.1038/s41467-022-29980-4)
Supplement: Supplementary file 3 — Reporting Summary [file 41467_2022_29980_MOESM3_ESM.pdf]

nature portfolio

Corresponding author(s): Yvain NICOLET

Last updated by author(s): Apr 1, 2022

Reporting Summary

Nature Portfolio wishes to improve the reproducibility of the work that we publish. This form provides structure for consistency and transparency in reporting. For further information on Nature Portfolio policies, see our Editorial Policies and the Editorial Policy Checklist.

Statistics

For all statistical analyses, confirm that the following items are present in the figure legend, table legend, main text, or Methods section.

n/a	Confirmed	
☒	☐	The exact sample size (*n*) for each experimental group/condition, given as a discrete number and unit of measurement
☒	☐	A statement on whether measurements were taken from distinct samples or whether the same sample was measured repeatedly
☒	☐	The statistical test(s) used AND whether they are one- or two-sided<br>*Only common tests should be described solely by name; describe more complex techniques in the Methods section.*
☒	☐	A description of all covariates tested
☒	☐	A description of any assumptions or corrections, such as tests of normality and adjustment for multiple comparisons
☒	☐	A full description of the statistical parameters including central tendency (e.g. means) or other basic estimates (e.g. regression coefficient) AND variation (e.g. standard deviation) or associated estimates of uncertainty (e.g. confidence intervals)
☒	☐	For null hypothesis testing, the test statistic (e.g. *F*, *t*, *r*) with confidence intervals, effect sizes, degrees of freedom and *P* value noted<br>*Give P values as exact values whenever suitable.*
☒	☐	For Bayesian analysis, information on the choice of priors and Markov chain Monte Carlo settings
☒	☐	For hierarchical and complex designs, identification of the appropriate level for tests and full reporting of outcomes
☒	☐	Estimates of effect sizes (e.g. Cohen's *d*, Pearson's *r*), indicating how they were calculated

Our web collection on statistics for biologists contains articles on many of the points above.

Software and code

Policy information about availability of computer code

Data collection	X-ray data were collected at the ESRF and SOLEIL beamlines using MXCuBE
Data analysis	X-ray data were processed using XDS (2019 release). X-ray models were refined using phenix_refine from the PHENIX suite (version 1.19.2-4158; including PHASER for molecular replacement as described in the manuscript). QM/MM calculations were performed using the program SCHORDINGER (Release 2020-04)

For manuscripts utilizing custom algorithms or software that are central to the research but not yet described in published literature, software must be made available to editors and reviewers. We strongly encourage code deposition in a community repository (e.g. GitHub). See the Nature Portfolio guidelines for submitting code & software for further information.

Data

Policy information about availability of data

All manuscripts must include a data availability statement. This statement should provide the following information, where applicable:

- Accession codes, unique identifiers, or web links for publicly available datasets
- A description of any restrictions on data availability
- For clinical datasets or third party data, please ensure that the statement adheres to our policy

Structure coordinates and diffraction data of the L-tyrosine-bound and the substrate-free TcThiH were deposited at the Protein Data Bank (http://www.pdb.org) under accession codes 7PD1 https://doi.org/10.2210/pdb7PD1/pdb and 7PD2 https://doi.org/10.2210/pdb7PD2/pdb, respectively. Source data are provided with this paper

Field-specific reporting

Please select the one below that is the best fit for your research. If you are not sure, read the appropriate sections before making your selection.

☒ Life sciences ☐ Behavioural & social sciences ☐ Ecological, evolutionary & environmental sciences

For a reference copy of the document with all sections, see nature.com/documents/nr-reporting-summary-flat.pdf

Life sciences study design

All studies must disclose on these points even when the disclosure is negative.

Sample size	Not applicable. X-ray structures were determined using single crystals
Data exclusions	No data was excluded
Replication	Not applicable. X-ray crystal structures were obtained from diffraction data from single crystals
Randomization	Not applicable. X-ray data were used to determine the crystal structures.
Blinding	Not applicable. Crystal structures were determined by molecular replacement. Rfree test set was applied by excluding randomly 5% of the data for refinement and were independantly compared with the calculated structure factors as always performed for crystal structure determination.

Reporting for specific materials, systems and methods

We require information from authors about some types of materials, experimental systems and methods used in many studies. Here, indicate whether each material, system or method listed is relevant to your study. If you are not sure if a list item applies to your research, read the appropriate section before selecting a response.

Materials & experimental systems

n/a	Involved in the study	
☒	☐	Antibodies
☒	☐	Eukaryotic cell lines
☒	☐	Palaeontology and archaeology
☒	☐	Animals and other organisms
☒	☐	Human research participants
☒	☐	Clinical data
☒	☐	Dual use research of concern

Methods

n/a	Involved in the study	
☒	☐	ChIP-seq
☒	☐	Flow cytometry
☒	☐	MRI-based neuroimaging
